# Association between intra-abdominal pressure and post-spinal hypotension during cesarean delivery: a prospective observational study

**DOI:** 10.3389/fmed.2026.1710374

**Published:** 2026-04-14

**Authors:** Rui Ma, Tao Xu, Shanshan Ye, Liqiang Zheng, Jing Zheng

**Affiliations:** 1 Department of Anaesthesiology, School of Medicine, The International Peace Maternity and Child Health Hospital, Shanghai Jiao Tong University, Shanghai, China; 2 Shanghai Key Laboratory of Embryo Original Diseases, Shanghai, China; 3 Clinical Research Center, the International Peace Maternity and Child Health Hospital, Shanghai Jiao Tong University School of Medicine, Shanghai, China; 4 Ministry of Education-Shanghai Key Laboratory of Children’s Environmental Health, Xinhua Hospital Affiliated to Shanghai Jiao Tong University School of Medicine, Shanghai, China

**Keywords:** cesarean delivery, hypotension, intra-abdominal hypertension, parturients, spinal anesthesia

## Abstract

**Background:**

Post-spinal hypotension remains a common challenge during cesarean delivery. This prospective study investigated the association between preanesthetic intra-abdominal pressure (IAP) and the risk of this complication.

**Methods:**

A total of 83 parturients undergoing elective cesarean delivery under spinal anesthesia were included. IAP was measured before anesthesia. The primary analysis used Poisson regression with robust standard errors to assess the association, reporting risk ratios for a clinically relevant 5-mmHg increase in IAP. A parsimonious model adjusted for body mass index (BMI) was used to ensure robustness. An exploratory receiver operating characteristic analysis with bootstrap internal validation was performed to describe the pressure’s discriminatory performance.

**Results:**

In the primary multivariable model, each 5-mmHg increase in IAP was associated with an approximately 3-fold higher risk of hypotension (adjusted relative risk (RR) = 2.88, 95% CI: 2.00–4.15, *p* < 0.001). Exploratory receiver operating characteristic (ROC) analysis yielded an area under the curve of 0.92; bootstrap validation indicated a corrected estimate of 0.919, and that a data-driven IAP cutoff was unstable, ranging from 12.5 to 13.5 mmHg. Using this exploratory threshold (≥12.5 mmHg), the unadjusted RR for hypotension was 7.79 (95% CI: 3.36–22.61).

**Conclusion:**

Higher baseline IAP is significantly associated with an increased risk of post-spinal hypotension during cesarean delivery. The association is robust when considering maternal body size. While an IAP threshold of approximately 12–14 mmHg may help identify parturients at greater risk, this finding is exploratory and requires external validation.

## 1 Introduction

Hypotension following spinal anesthesia for cesarean delivery remains a common clinical challenge, with reported incidences of 50–70% (1). Current predictive models often focus on assessing baseline autonomic tone, which may be influenced by maternal anxiety and other transient factors (2–5). However, a key component of post-spinal hypotension in parturients is the mechanical compression of the inferior vena cava (IVC) by the gravid uterus. There is a need for a predictor that more directly reflects this physical component of impaired venous return.

Intra-abdominal pressure (IAP), defined as the steady-state pressure within the abdominal cavity, increases progressively during pregnancy (6, 7). Elevated IAP is a recognized surrogate for the degree of mechanical compression exerted on the IVC (8, 9). Although ultrasound assessment of the IVC is feasible, routine preoperative measurements in the obstetric setting are not standardized. In contrast, IAP can be measured conveniently via a standard urinary catheter, offering a potential practical advantage.

We, therefore, hypothesized that higher preanesthetic IAP would be associated with an increased risk and greater severity of post-spinal hypotension. This pilot study aimed to evaluate this association and to explore the discriminatory ability of IAP in this context.

## 2 Materials and methods

This prospective observational study was approved by the Medical Research Ethics Committee of the International Peace Maternity and Child Health Hospital, Shanghai, China (Chairperson Zhiwei Liu) on September 19, 2023 (GKLW-A-2023-035-01). The study was designed in accordance with applicable STROBE guidelines. Written informed consent was obtained from all participants after recruitment.

### 2.1 Inclusion and exclusion criteria

A total of 83 full-term parturients with singleton pregnancies, aged 20–40 years, with heights ranging from 156 to 170 cm, an American Society of Anesthesiologists physical status classification of II, and scheduled for elective cesarean delivery under combined spinal–epidural (CSE) anesthesia at the International Peace Maternity and Child Health Hospital were recruited between 22 November 2023 and 26 February 2024.

The exclusion criteria were as follows: contraindications to spinal anesthesia, allergy to ropivacaine, preeclampsia or hypertension, underlying valvular disease detected by preoperative echocardiography, multiple pregnancies, body mass index (BMI) > 35 kg/m^2^, active labor, emergency cesarean delivery, and parturient refusal. Parturients with difficult epidural punctures and an anesthesia level below T6 before surgery were also withdrawn after enrollment.

### 2.2 Study design

All parturients fasted overnight and were administered 500 mL of crystalloid solution through an 18-G cannula within 30 min of entering the operating room. Routine monitoring included non-invasive blood pressure measurements, electrocardiography, and pulse oximetry, which were performed continuously. Consecutive systolic blood pressure (SBP) and heart rate (HR) values were recorded in the supine position every minute; the average of the first two readings was treated as the baseline. Parturients with an SBP of >140 mmHg were excluded due to suspected hypertension.

### 2.3 Measurement of IAP

As per routine practice, a transurethral 16-Fr Foley catheter was inserted to drain the bladder before anesthesia. Pressure measurements were recorded in the supine position using an intravesical pressure measurement system at the end of expiration when the abdominal wall was relaxed. This procedure followed the method described in the World Society of the Abdominal Compartment Syndrome (WSACS) guidelines (10). The pressure measurement kit (Exadyn Combitrans Monitoring Set; B. Braun Melsungen AG, Germany) was connected to a monitor (IntelliVue MP50; Philips Medical Systems Boeblingen GmbH, Germany) to determine the IAP value. The mid-axillary level in the supine position, with a bladder inflation volume of 25 mL, was considered the zero reference. The first steady value was recorded as the IAP value. IAP and other maternal characteristics were measured and recorded by the same trained researcher; the anesthesiologist and the parturients were blinded to these measurements.

### 2.4 Procedure for anesthesia induction

An anesthesiologist with over 15 years of experience performed the CSE puncture at the L3–L4 interspace with the parturient in the right lateral position. A 17-G Tuohy needle was used, followed by the insertion of a 27-G Whitacre needle through the Tuohy needle to access the subarachnoid space. Upon identification of cerebrospinal fluid, 15 mg of 0.5% plain ropivacaine was injected. An epidural catheter was then threaded 4 cm into the epidural space. Subsequently, the parturient was repositioned to the supine position, and a 30° wedge was placed under the right hip. The upper anesthesia level was assessed by a pinprick; parturients whose anesthesia level remained below T6 at the beginning of surgery were withdrawn from the study. SBP and HR were recorded every minute for 12 min before delivery and then every 5 min until the end of surgery using a GE B125M patient monitor (GE Healthcare, Milwaukee, WI, United States).

Hypotension was defined as a drop in SBP of >20% from the baseline value (11, 12). A rescue dose of 50 μg of phenylephrine was administered upon the occurrence of hypotension. Bradycardia, defined as HR < 50 beats/min, was treated with intravenous atropine (0.5 mg).

After delivery, Apgar scores at 1 and 5 min, along with neonatal body weight, were recorded. An obstetrician immediately collected 1 mL of umbilical artery (UA) blood, and blood gas analysis was performed using a blood gas analyzer (Edan i15 Blood Gas Analyzer; ICEN, Guangdong, China) with an iSTAT BG10 test cartridge.

The primary outcome was the association between IAP and post-spinal hypotension during cesarean delivery. Maternal and obstetric characteristics included age, height, weight, BMI, gravidity, parity, amniotic fluid index, expected fetal weight by ultrasound within 1 week before cesarean delivery, uterine height, abdominal circumference, total doses of phenylephrine and atropine administered, upper anesthesia level, incision–delivery interval, neonatal body weight, Apgar scores at 1 and 5 min, pH and base excess of UA blood, and maternal adverse symptoms.

### 2.5 Sample size calculation and statistical analysis

From clinical experience, the probability of hypotension occurring during cesarean delivery after administering 15 mg of 0.5% plain ropivacaine into the subarachnoid space is approximately 50% (10). Based on an *a priori* power analysis (PASS 2021), a sample size of 86 parturients was required to detect an area under the curve (AUC) of 0.8 for IAP in predicting post-spinal hypotension (*α* = 0.05, CI width = 0.2), considering a 10% dropout rate.

All analyses were conducted using R 4.4.1 (R Foundation for Statistical Computing, Vienna, Austria). Data cleaning and manipulation were performed using the tidyverse package. The tableone package was used to summarize baseline characteristics. Continuous variables are presented as mean ± standard deviation or median (P25, P75), and categorical variables as percentages. The distribution of variables was assessed using the Kolmogorov–Smirnov test.

### 2.6 Model building strategy and variable selection

Given the limited number of hypotension events (*n* = 43), we adopted a parsimonious modeling strategy to prevent overfitting and to address potential conceptual collinearity among anthropometric measures. Although univariable analyses identified several body size measures (e.g., abdominal circumference and BMI) as significantly associated with the outcome, BMI was selected as the primary adjustment variable in the core model for two reasons. First, BMI represents a fundamental and integrative measure of overall maternal body size and composition; second, adjusting for BMI minimizes conceptual collinearity with IAP, as BMI is a systemic measure less likely to capture the same localized physiological information as abdominal circumference, thereby allowing for a clearer assessment of IAP’s specific association with hypotension.

### 2.7 Primary analysis: association between IAP and hypotension

The primary analysis assessed the association between IAP (modeled as a continuous variable, per 1- and per 5-mmHg increase) and the risk of post-spinal hypotension using Poisson regression with robust standard errors (via the sandwich and lmtest packages). Our pre-specified core model adjusted for BMI only. The results are presented as relative risks (RRs) with 95% confidence intervals (CIs).

### 2.8 Secondary and exploratory analyses

1. Descriptive Univariable Analyses: Univariable Poisson regressions (with robust standard errors) were performed for all candidate variables to describe their unadjusted associations with hypotension. These results are presented in a descriptive context.
2. Comprehensive Multivariable Model: An exploratory model including all covariates (IAP, weight, age, BMI, etc.) was fitted. The results are provided in Supplementary material to ensure transparency while acknowledging its limited stability due to the events-per-variable ratio.
3. Relationship between IAP and SBP decrease: To visually explore the relationship between baseline IAP and the magnitude of SBP decrease, a scatterplot was constructed and supplemented with Spearman’s rank correlation coefficient (*ρ*).
4. ROC and Cutoff Analysis: An exploratory receiver operating characteristic (ROC) analysis was performed using the pROC package. To correct for optimism bias, bootstrap internal validation was performed on 1,000 replicates. The bias-corrected AUC was reported. All findings from this analysis are explicitly framed as exploratory and hypothesis-generating.
5. Analysis Using an IAP Cutoff: In a secondary *post-hoc* analysis, IAP was dichotomized using the exploratory cutoff. The results are presented separately with a clear disclaimer about the statistical limitations of dichotomization.
6. Repeated Measures Analysis: Differences in systolic arterial pressure and heart rate over time were compared between groups using linear mixed-effects models (via the lme4 package), incorporating a random subject intercept to account for within-individual correlation.

## 3 Results

### 3.1 Baseline characteristics and incidence of hypotension

**Figure 1:**
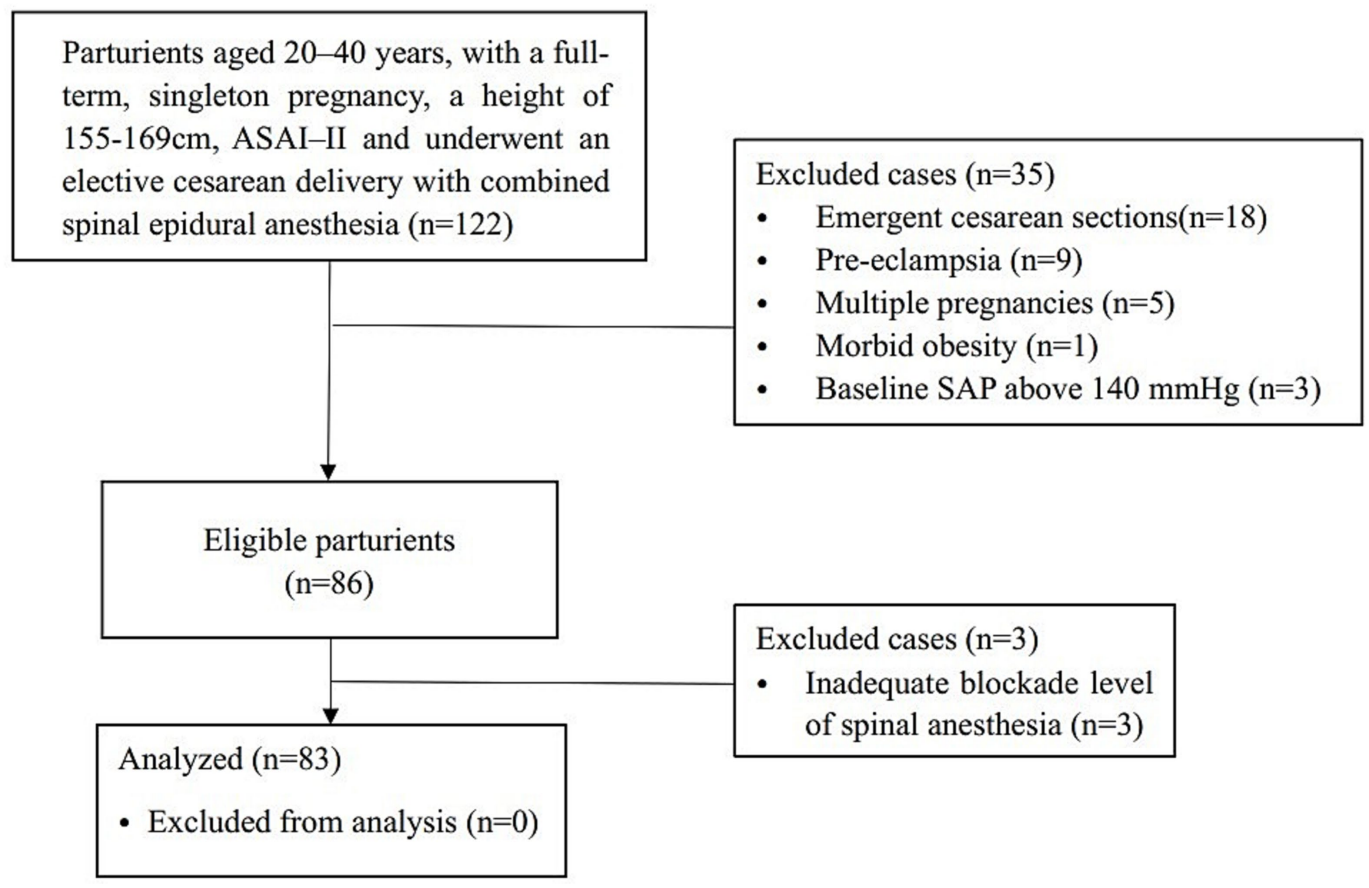
Flowchart of the study.

A total of 122 parturients were screened. After applying the predefined exclusion criteria (see Figure 1), 86 parturients were eligible and underwent spinal anesthesia. Among these, three were excluded due to inadequate anesthesia blockade, resulting in a final analytic sample of 83 parturients (Figure 1). The maternal and obstetric characteristics of parturients, categorized into hypotension (*n* = 43) and non-hypotension (*n* = 40) groups, are presented in Table 1. The mean IAP before anesthesia induction was higher in the hypotension group than in the non-hypotension group (14.9 ± 2.3 mmHg vs. 10.5 ± 2.3 mmHg, *p* < 0.001). The distribution of measured IAP showed differences between the two groups. Other characteristics that also significantly differed between the groups included height, BMI, expected fetal weight by ultrasound, uterine height, and abdominal circumference.

**Table 1:** Maternal and obstetric characteristics of all parturients and categorized into hypotension and non-hypotension groups.

Variables	Non-hypotension (*N* = 40)	Hypotension (*N* = 43)	*p-*value
Age (year)	33.7 ± 3.5	31.7 ± 3.7	0.012
Height (cm)	163.4 ± 3.0	161.2 ± 3.9	0.005
Weight (kg)	68.7 ± 9.0	72.2 ± 9.4	0.090
BMI (kg/m^2^)	25.7 ± 3.0	27.8 ± 3.3	0.004
Gestational week (wk)	38.51 ± 0.99	38.77 ± 0.92	0.232
Gravidity	1 (1, 2)	2 (1, 3)	0.050
Parity	0 (0, 1)	0 (0, 1)	0.570
AFI (mm)	127.1 ± 33.0	131.1 ± 31.8	0.574
Expected fetal weight (g)	3140.6 ± 421.9	3398.1 ± 426.1	0.007
Uterine height (cm)	35.7 ± 3.6	38.0 ± 3.8	0.005
Abdominal circumference (cm)	93.9 ± 6.7	98.5 ± 7.0	0.003
IAP (mmHg)	10.5 ± 2.3	14.9 ± 2.3	<0.001
IAP, *n* (%)			<0.001
<12 mmHg	28 (70)	2 (4.7)	
12–15 mmHg	10 (25)	24 (55.8)	
>15 mmHg	2 (5)	17 (39.5)	

Values are mean ± SD or median (IQR). IQR, interquartile range; SD, standard deviation; BMI, body mass index; AFI, amniotic fluid index; IAP, intra-abdominal pressure.

### 3.2 Primary analysis: association of IAP with hypotension risk

#### 3.2.1 Univariable associations

In univariable Poisson regression analyses with robust standard errors, several factors were significantly associated with an increased risk of post-spinal hypotension. Both IAP (*p* < 0.001) and abdominal circumference (*p* < 0.001) showed strong associations, and BMI was also significantly associated (*p* = 0.001). The complete univariable results are presented in Supplementary Table S1 for descriptive context.

#### 3.2.2 Primary multivariable model

Given the limited number of hypotension events (*n* = 43), a pre-specified parsimonious multivariable model was used to ensure stability and interpretability. After adjustment for BMI, each 5-mmHg increase in baseline IAP was associated with an approximately threefold higher risk of post-spinal hypotension (adjusted RR = 2.88, 95% CI: 2.00–4.15, *p* < 0.001). The results of the primary multivariable Poisson regression model are presented in Table 2.

**Table 2:** Results of the multivariable Poisson regression analysis for factors associated with post-spinal hypotension.

Variable	Adjusted RR (95%CI)	*p*-value
IAP		
Per 1-mmHg increase	1.24 (1.15–1.33)	<0.001
Per 5-mmHg increase	2.88 (2.00–4.15)	<0.001
BMI		
Per 1-kg/m^2^ increase	1.02 (0.97–1.08)	0.485

This parsimonious model includes IAP and BMI. IAP is expressed per 1- and 5-mmHg increase to reflect a clinically meaningful change. IAP, intra-abdominal pressure; BMI, body mass index.

**Figure 2:**
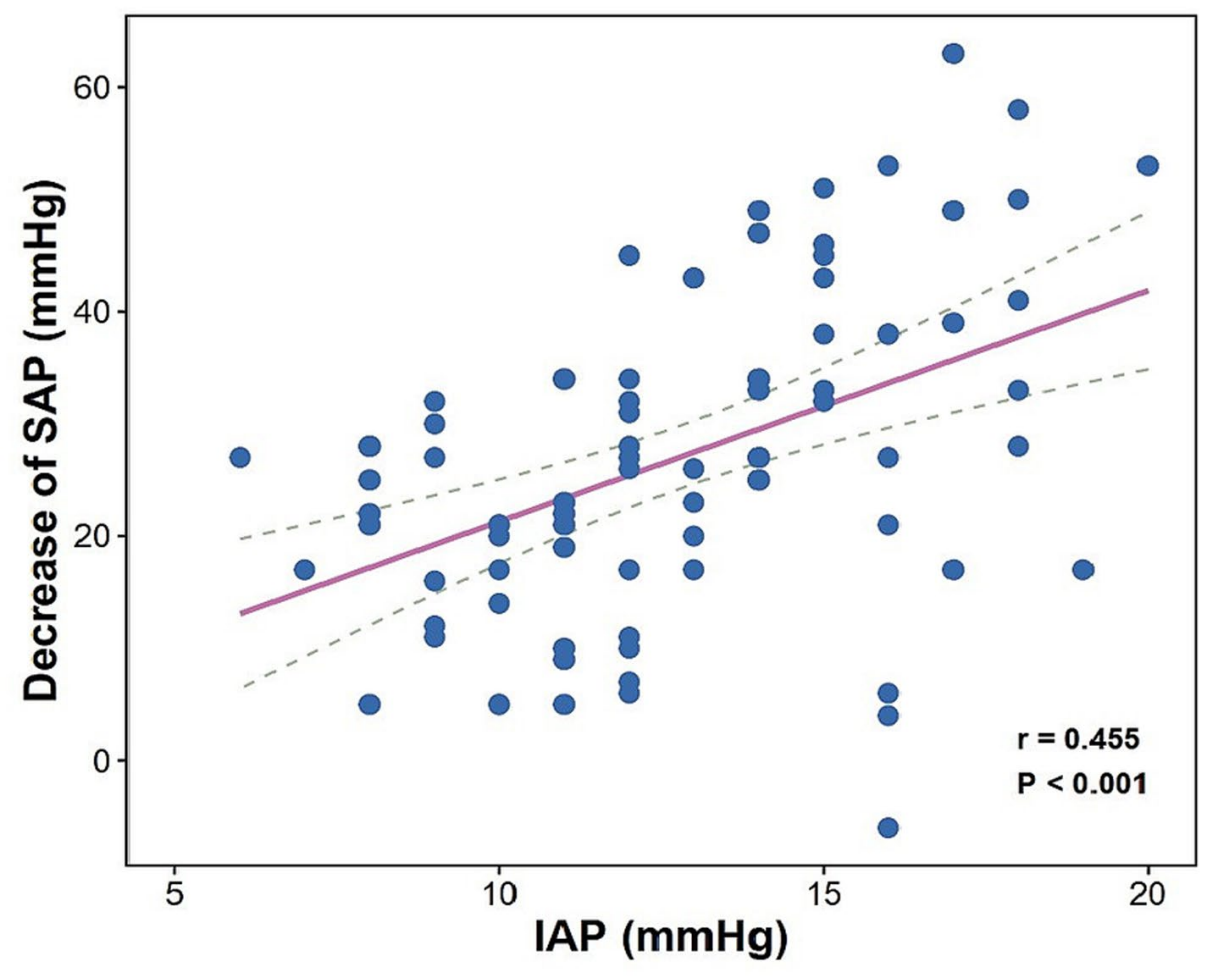
Correlation between IAP and a decrease in SBP. The decrease in SBP = baseline SBP–lowest SBP. The solid line represents the linear regression line, and the dotted lines represent the 95% confidence intervals. SBP, systolic blood pressure; IAP, intra-abdominal pressure.

In line with the regression findings, preliminary exploratory analysis indicated a moderate positive correlation between higher baseline IAP and a greater decrease in SBP from baseline (Spearman’s *ρ* = 0.455, *p* < 0.001) (see Figure 2).

#### 3.2.3 Exploratory comprehensive model

An exploratory model including all covariates (IAP, age, BMI, etc.) yielded results consistent with the primary model, although the confidence intervals were wider. The full results are presented in Supplementary Table S2.

### 3.3 Secondary and exploratory analyses

#### 3.3.1 ROC analysis with internal validation

The exploratory ROC analysis indicated that IAP had discriminatory ability within this cohort, with an area under the curve (AUC) of 0.92 (95% CI: 0.85–0.99). Bootstrap internal validation (1,000 replicates) indicated an optimism-corrected AUC of 0.919.

The bootstrap internal validation showed that the data-driven optimal cutoff of 12.5 mmHg was not a fixed-point estimate. The 95% percentile interval of the bootstrap-derived cutoffs ranged from 12.5 to 13.5 mmHg (see Supplementary Figure S1), indicating expected variability in this estimate when derived from a single cohort of the present sample size.

This cutoff is hypothesis-generating and requires external validation before any clinical application.

#### 3.3.2 Analysis using the exploratory IAP cutoff

When IAP was *post-hoc* dichotomized at the exploratory cutoff of 12.5 mmHg, parturients in the high-IAP group (≥12.5 mmHg) had a markedly higher risk of post-spinal hypotension in the unadjusted analysis (RR = 7.79, 95% CI: 3.36–22.61, *p* < 0.001). All subsequent analyses using this dichotomization are presented as exploratory findings.

#### 3.3.3 Repeated measures of hemodynamics (linear mixed-effects model)

The linear mixed-effects model revealed a significant interaction between the IAP group and time on SBP (*p* < 0.001), indicating divergent hemodynamic trajectories.

*Post-hoc* pairwise comparisons at each time point showed that the SBP in the high-IAP group was significantly lower than in the low-IAP group at 2, 3, 4, 5, 6, 11, and 12 min after induction, with the largest difference observed at 3 min (mean difference: 17.62 mmHg, SE: 2.6, *p* < 0.0001). No significant differences were found in heart rate trajectories.

#### 3.3.4 Clinical outcomes from the exploratory IAP group

Exploratory comparisons of clinical outcomes between the IAP groups are detailed in Supplementary Table S3. In brief, the high-IAP group (≥12.5 mmHg) had a significantly higher incidence of hypotension(*p* < 0.001), required higher doses of phenylephrine (*p* < 0.001), and had a higher incidence of nausea (*p* = 0.005). Additionally, the high-IAP group exhibited a greater fetal birth weight (*p* = 0.008)

## 4 Discussion

This pilot study investigated the association between IAP and the risk of hypotension following spinal anesthesia for cesarean delivery. Our primary and most robust finding is that higher baseline IAP is independently associated with a significantly increased risk of post-spinal hypotension, even after adjusting for BMI. To delineate the intrinsic relationship, we used a reactive vasopressor strategy and observed the natural course of hemodynamic changes.

### 4.1 Interpretation of findings

The results are consistent with the primary hypothesis that elevated IAP may exacerbate post-spinal hypotension through augmented mechanical compression of the IVC. In the context of sympathetic blockade, this heightened compression likely contributes to a more pronounced reduction in venous return (13, 14). This pathophysiological link is supported by studies from other clinical contexts, which demonstrate that increased IAP can reduce cardiac output via IVC compression (8, 9, 15–17).

We also considered the alternative perspective that a higher IAP, often correlating with increased fundal height, might influence cerebrospinal fluid dynamics and lead to a more extensive sensory blockade (18, 19). In our cohort, rigorously assessed for sensory block height, no statistically significant difference was found between high- and low-IAP groups. This suggests that differential sensory blockade is not the primary driver of the observed hemodynamic differences in this controlled population; it remains a physiologically plausible factor. Therefore, our data lend greater support to the hemodynamic mechanism of IVC compression as a probable key contributor to the association. Future research incorporating detailed hemodynamic monitoring is needed to elucidate the interaction between these mechanisms.

### 4.2 Clinical implications

The clinical relevance of pre-anesthetic IAP assessment lies in its potential for enhanced physiological risk stratification, providing an additional data point beyond standard anthropometric measures. For parturients with lower IAP, this might support a more conservative approach to routine prophylactic vasopressor administration. For those with elevated IAP (e.g., in the exploratory range of ≥12–14 mmHg), this information could contribute to a more vigilant anesthetic strategy. This might include intensified hemodynamic monitoring, patient counseling, consideration of techniques allowing for more gradual sympathetic blockade, and efforts to minimize the anesthesia-to-delivery interval (12, 20, 21).

### 4.3 Study strengths and limitations

Key strengths include the prospective design, the use of a parsimonious statistical model to ensure robust inference, and the operational feasibility of IAP measurement. Bootstrap validation of the ROC analysis provided an objective assessment of the optimism in our exploratory cutoff value.

As a pilot investigation, this study has several limitations. First, bootstrap validation indicated that the exploratory IAP cutoff value was not a fixed point but ranged from 12.5 to 13.5 mmHg. While this range is clinically narrow, it confirms the statistical instability inherent in deriving a threshold from a single cohort. Thus, it should be interpreted as an exploratory range requiring external validation. Second, the institutional protocol at the time of the study deliberately avoided routine prophylactic vasopressors to observe the natural incidence of hypotension. This may limit the generalizability of our absolute incidence rates to contemporary practice settings, where prophylactic regimens are standard. However, it does not invalidate the observed associations between baseline physiological variables and the risk of hypotension. Third, the single-center design and strict exclusion criteria enhance internal validity but necessitate validation in broader, more diverse obstetric populations. Fourth, although we adopted a parsimonious modeling strategy to mitigate overfitting, the events-per-variable ratio in our primary model remained modest. Therefore, some degree of residual overfitting cannot be entirely excluded, and our findings warrant confirmation in larger cohorts.

## 5 Conclusion

In conclusion, this study provides evidence that higher baseline IAP is significantly associated with an increased risk of post-spinal hypotension during cesarean delivery. The association is robust to adjustment for BMI. An IAP value in the 12–14 mmHg range may serve as an exploratory indicator of higher risk, but this threshold requires external validation. Our findings support the integration of IAP as a physiological variable in future larger-scale studies to refine multivariable risk prediction models and guide personalized hemodynamic management.

## Data Availability

The raw data supporting the conclusions of this article will be made available by the authors, without undue reservation.

## Supplementary material

Supplementary Figure S1 Exploratory ROC analysis and bootstrap validation for IAP. **(A)** ROC curve depicting the ability of baseline IAP to discriminate parturients who developed post-spinal hypotension in this cohort. The area under the curve (AUC) was 0.92 (95% CI: 0.85–0.99). Internal validation via bootstrap resampling (1,000 replicates) yielded an optimism-corrected AUC of 0.919. **(B)** Distribution of the optimal IAP cut-off value identified across 1,000 bootstrap samples. The vertical dashed line indicates the original cut-off derived from the full cohort (12.5 mm Hg). The shaded region represents the 95% percentile interval (12.5–13.5 mm Hg), illustrating the variability of this data-driven estimate and underscoring its exploratory nature. ROC, receiver operating characteristic; IAP, intra-abdominal pressure.
