# Supplementary figures and images for: Association between intra-abdominal pressure and post-spinal hypotension during cesarean delivery: a prospective observational study
Source: Front Med (Lausanne). 2026 Apr 14;13:1710374. doi: 10.3389/fmed.2026.1710374 (PMC13121375; doi:10.3389/fmed.2026.1710374)

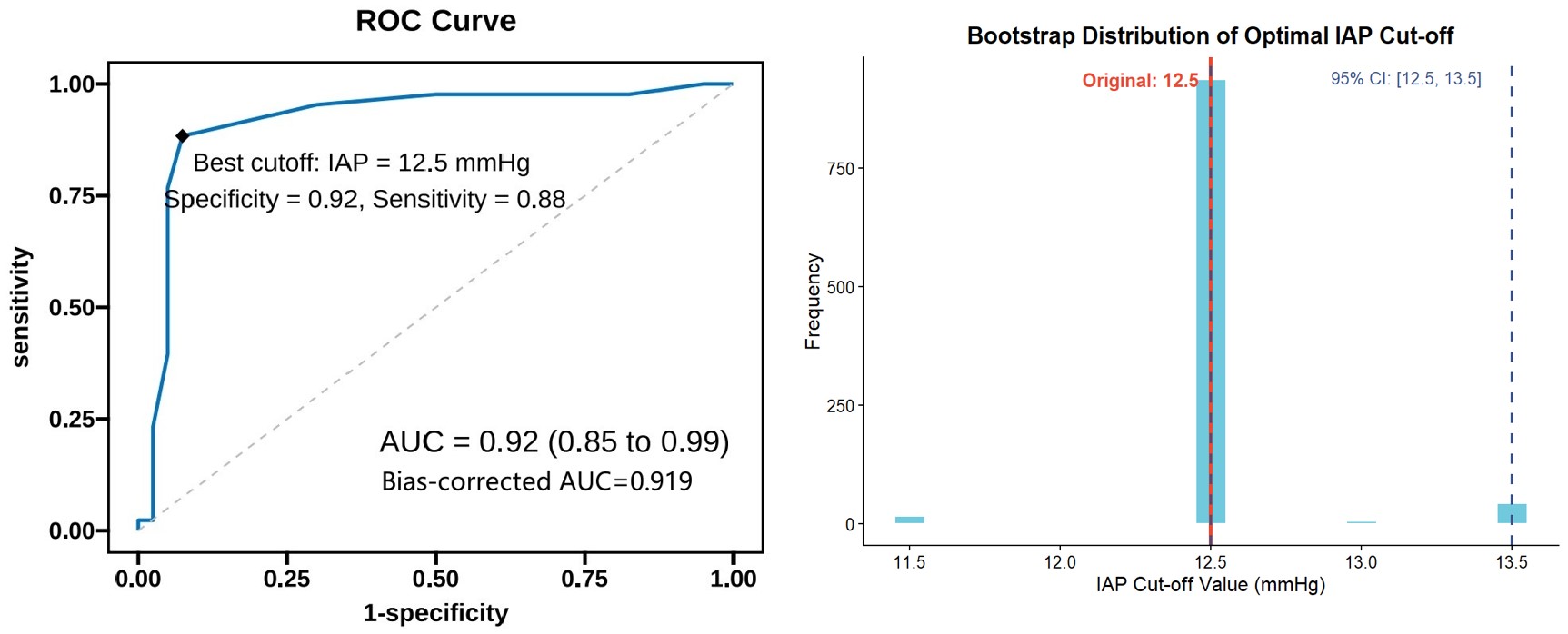

Supplement: Supplementary Figure S1 — Exploratory ROC analysis and bootstrap validation for IAP. (A) ROC curve depicting the ability of baseline IAP to discriminate parturients who developed post-spinal hypotension in this cohort. The area under the curve (AUC) was 0.92 (95% CI: 0.85–0.99). Internal validation via bootstrap resampling (1,000 replicates) yielded an optimism-corrected AUC of 0.919. (B) Distribution of the optimal IAP cut-off value identified across 1,000 bootstrap samples. The vertical dashed line indicates the original cut-off derived from the full cohort (12.5 mm Hg). The shaded region represents the 95% percentile interval (12.5–13.5 mm Hg), illustrating the variability of this data-driven estimate and underscoring its exploratory nature. ROC, receiver operating characteristic; IAP, intra-abdominal pressure. [file Image_1.jpeg]
