# Supplementary material for: Association between intra-abdominal pressure and post-spinal hypotension during cesarean delivery: a prospective observational study
Source: Front Med (Lausanne). 2026 Apr 14;13:1710374. doi: 10.3389/fmed.2026.1710374 (PMC13121375; doi:10.3389/fmed.2026.1710374)
Supplement: Supplementary file 2 [file Table_1.docx]

**Table S1** Univariable analysis of risk factors associated with hypotension

| Variable | RR (95% CI) | p- value |
| --- | --- | --- |
| IAP | 1.24 (1.16-1.33) | <0.001 |
| BMI | 1.09 (1.04-1.15) | 0.001 |
| Age | 0.93 (0.89-0.98) | 0.011 |
| gravidity | 1.14 (0.99-1.30) | 0.068 |
| Gestational week | 1.22 (0.99-1.51) | 0.066 |
| Expect weight | 1.00 (1.00-1.00) | 0.002 |
| Uterine height | 1.08 (1.00-1.13) | 0.005 |
| Abdominal circumference | 1.04 (1.00-1.07) | <0.001 |

IAP: Intra-abdominal Pressure; BMI: body mass index.

**Table S2** Multivariable analysis of risk factors associated with hypotension

| Variable | RR (95% CI) | p- value |
| --- | --- | --- |
| IAP | 1.26 (1.13-1.40) | <0.001 |
| Age | 1.00(0.92-1.08) | 0,942 |
| BMI | 1.07 (0.97-1.17) | 0.170 |
| gravidity | 1.19 (1.00-1.40) | 0.045 |
| Gestational week | 1.00(0.82-1.23) | 0.967 |
| Expect weight | 1.00 (1.00-1.00) | 0.438 |
| Uterine height | 1.02 (0.97-1.07) | 0.538 |
| Abdominal circumference | 0.97 (0.91-1.02) | 0.195 |

IAP: Intra-abdominal Pressure; BMI: body mass index.

**Table S3** Maternal and neonatal observations between normal IAP (IAP<12.5mmHg) and high IAP (IAP≥12.5mmHg) groups

| Variables | Normal IAP  (n = 42) | High IAP  (n = 41) | P value |
| --- | --- | --- | --- |
| Post-spinal hypotension, n (%) | 5 (11.9) | 38 (92.7) | <0.001 |
| Phenylephrinedose (µg) | 0 (0, 0) | 100 (50,100) | <0.001 |
| Phenylephrine times | 0 (0, 0) | 1 (1, 2) | 0.005 |
| Dyspnoea, n (%) | 0 (0.0) | 4 (9.8) | 0.055 |
| Nausea, n (%) | 0 (0.0) | 7 (17.1) | 0.005 |
| Induction-delivery interval (min) | 9.7±1.4 | 10.0±2.0 | 0.450 |
| The upper sensory level | 4 (4, 4) | 4 (4, 4) | 0.337 |
| Baby birth weight (g) | 3221.8±493.7 | 3513.7±480.8 | 0.008 |
| Apgar scores at 1 minute | 10 (10, 10) | 10 (10, 10) | 0.494 |
| Apgar scores at 5 minutes | 10 (10, 10) | 10 (10, 10) | N/A |
| pH | 7.34±0.05 | 7.33±0.03 | 0.342 |
| BE (mmol/L) | -2.68±1.48 | -2.86±1.74 | 0.681 |

Values are mean±SD, number (percentage), or median (IQR).

IQR: interquartile range; SD: standard deviation; BE: base excess.
